# Exploring the Long-Term Effect of Artificial Sweeteners on Metabolic Health

**DOI:** 10.7759/cureus.70043

**Published:** 2024-09-23

**Authors:** Meenatchi M, Chitra Vellapandian

**Affiliations:** 1 Department of Pharmacology, Sri Ramaswamy Memorial (SRM) College of Pharmacy, SRM Institute of Science and Technology, Kattankulathur, IND

**Keywords:** artificial sweeteners, dysbiosis, health risks, metabolic, obesity, type 2 diabetes

## Abstract

Artificial sweeteners (ASs) are widely used as low-calorie sugar substitutes for managing conditions like diabetes and obesity, but recent evidence suggests their health effects may be more complex than previously understood. High consumption has been associated with increased risks of metabolic disorders, cardiovascular diseases, certain cancers, and, somewhat paradoxically, weight gain, adverse pregnancy outcomes, and potential risks for individuals with low seizure thresholds. Studies, including the Women's Health Initiative, have linked artificially sweetened beverages to an elevated risk of stroke, coronary heart disease, and mortality, independent of established risk factors. Concerns extend to gut health, where ASs like saccharin have been linked to inflammatory bowel diseases, gut microbiota disruption, increased intestinal permeability, and dysbiosis, leading to metabolic disturbances such as impaired glucose tolerance, insulin resistance, and heightened systemic inflammation. These disruptions reduce the production of short-chain fatty acids crucial for insulin sensitivity, further contributing to the development of metabolic disorders like type 2 diabetes mellitus. Given these potential health risks, this review underscores the need for cautious use, informed consumer choices, and stringent regulatory oversight, while emphasizing the necessity for further research to elucidate long-term health effects and develop strategies to mitigate these risks.

## Introduction and background

Artificial sweeteners (ASs), widely used as low-calorie sugar substitutes, play a significant role in managing conditions like diabetes and obesity, but recent studies reveal a more complex health impact. High consumption of these sweeteners is linked to a 30% increased risk of metabolic problems, cardiovascular disease, and several types of cancer [1]. For example, the Women's Health Initiative found an independent relationship between excessive consumption of artificially sweetened beverages and increased risks of stroke, coronary heart disease, and mortality. The acceptable daily intake (ADI) for aspartame is 50 mg/kg body weight per day in the United States and 40 mg/kg in the European Union, while for saccharin, the ADI is set at 5 mg/kg body weight [2]. Despite their role in weight management, ASs may paradoxically lead to weight gain. Specific concerns include aspartame, which is associated with neurotoxicity and cancer risks, especially for those with phenylketonuria [3]; sucralose, which alters gut flora and may lead to glucose metabolism issues and insulin resistance; and saccharin, linked to gut microbiota disruption and inflammatory bowel diseases (IBDs) [4]. Acesulfame-K raises concerns about metabolic disruption and cancer risk [5]. Furthermore, ASs like sucralose and aspartame can disrupt insulin signaling, inducing hyperinsulinemia and insulin resistance by altering glucose transporter expression and insulin receptor sensitivity. They also affect the gut microbiota, with saccharin and sucralose linked to dysbiosis, glucose intolerance, and systemic inflammation, contributing to metabolic syndrome [4]. Concerns about genotoxicity and the potential impact of sweeteners like acesulfame-K on gut flora and cancer risk have been raised by regulatory bodies like the European Food Safety Authority (EFSA) [5]. Given these health risks, consumers and healthcare professionals must remain informed to make healthier dietary choices.

## Review

Types of ASs

**Figure 1:**
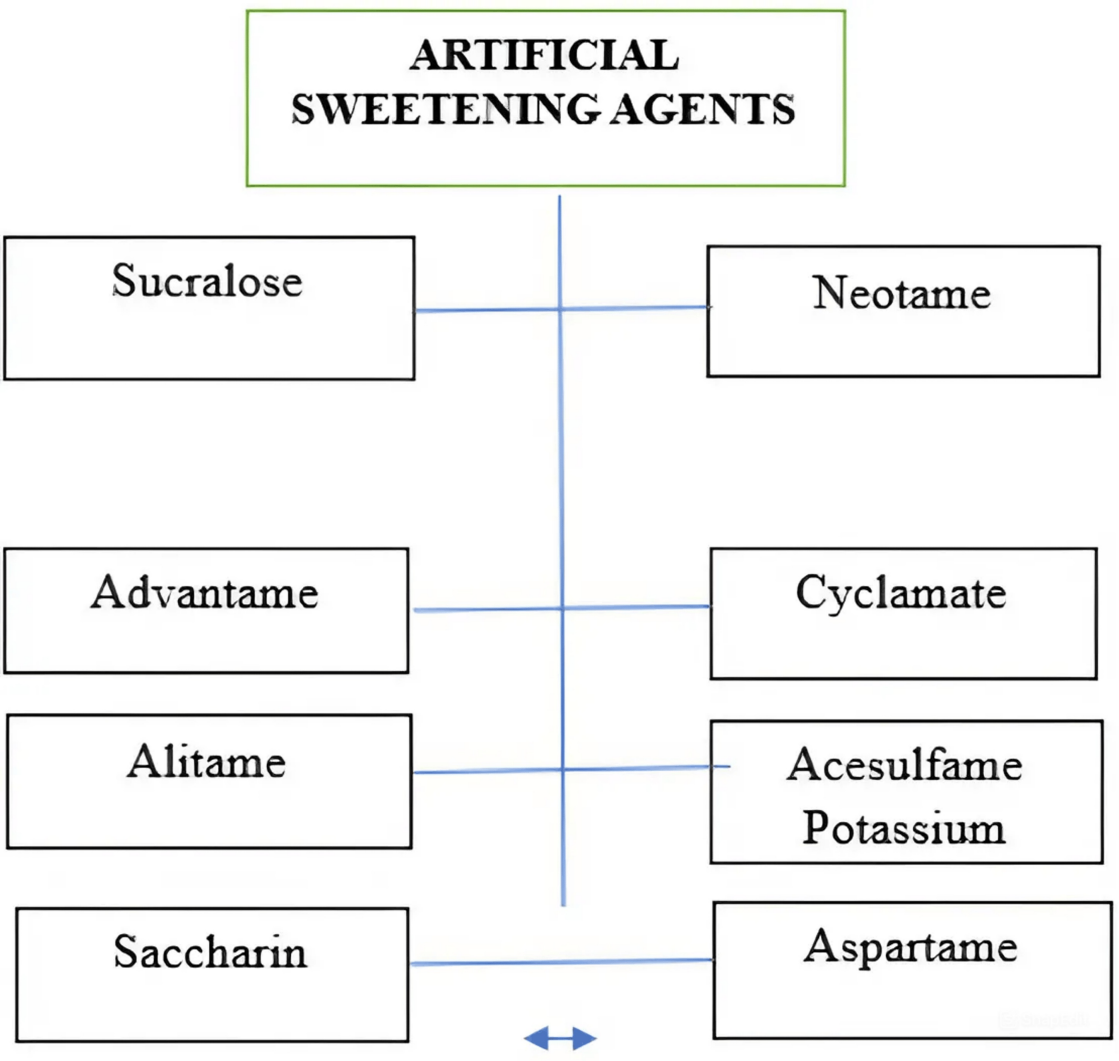
Types of ASs ASs: artificial sweeteners This image was illustrated by the author Meenatchi M

Various types of ASs are listed in Figure 1.

ASs are described in terms of their relative sweetness compared to sugar, well-known brand names, and ADI values, expressed in milligrams per kilogram of body weight per day. These sweeteners are commonly used in products like tabletop sweeteners, beverages, and baked goods. Potential health risks, including concerns about metabolism, cancer, and gut flora disruption, are also addressed. The ADI values indicate the safe daily consumption levels over a lifetime, as illustrated in Table 1.

**Table 1:** Comprehensive guide to ASs: uses, risks, and ADI ASs: artificial sweeteners; ADI: acceptable daily intake; PKU: phenylketonuria

ASs	More sweetener than sugar	Brand names	ADI (mg/kg/day)	Uses	Risk factor	Study
Aspartame	200	NutraSweet	50	Extensively utilized in tabletop sweeteners, chewable vitamins, confectionery mixes, chewing gum, and beverages	PKU patients' phenylalanine sensitivity, high doses' possible harm to the liver, and concerns about neurotoxicity and cancer risks all surround safety	Ali et al. [6]
Acesulfame-K	200	Sweet one	15	Used as a tabletop sweetener and frequently mixed with other sweeteners in soft drinks, baked goods, candies, and chewing gum	Worries regarding adverse effects on metabolism, possible cancer risk, and long-term safety	Ali et al. [6]
Saccharin	600	Sweet N’ low	5	Frequently present in chewing gum, canned fruit, baked foods, tabletop sweeteners, and diet sodas	Early research showed a potentially disrupted gut flora and a risk of bladder cancer, which has since been completely debunked	Mahmood and Al-Juboori [7]
Sucralose	300	Splenda	5	Used in tabletop sweeteners, dairy products, baked items, frozen sweets, and beverages	Changes in gut flora, potential to affect insulin release, potential to modify glucose metabolism, and weight gain in certain studies	Gujral et al. [8]
Neotame	8,000	Neotame	2	Used as a general-purpose sweetener in dairy products, baked goods, and beverages; authorized for usage other than in meat and poultry	Possible harm to the liver from overindulgence, weight fluctuations, minor headaches, and appetite loss	Gibbons et al. [9]
Cyclamate	30	-	1	Banned in the United States but utilized in other nations; this sweetener is used in beverages, baked products, sweets, and tabletop confections	Potentially harmful effect on male fertility and connection to bladder cancer (illegal in several countries)	Kramer [10]
Alitame	2,000	-	0-1	Less prevalent because of regulatory obstacles; used in dairy products, baked items, chewing gum, and beverages	Relatively less information is available on potential liver damage than other sweeteners	Ali et al. [6]
Advantame	37,000	-	5	Advantame 37,000-5 is suitable for high temperatures and low pH, and is used in drinks, gum, yogurt, cakes, and powdered drink mixes	Restricted long-term evidence, but recent studies have not found any substantial toxicological consequences; generally regarded as safe	Ali et al. [6]

The properties of different sweeteners, including their physical attributes, stability, and solubility, are explored. Their safety and metabolism are also considered, focusing on how they are processed in the body and potential health risks. Research findings provide key insights into their safety, health effects, and effectiveness, with references linked to the relevant studies cited in the text, as illustrated in Table 2.

**Table 2:** Properties, safety, and metabolism of various sweeteners NAC: N-acetylcysteine; IARC: International Agency for Research on Cancer; NTP: National Toxicology Program

Sweeteners	Properties	Safety and metabolism	Toxicology and pathophysiology	Study
Aspartame	White, odorless powder; dissolves in water; highest stability at pH 4.3	Breaks down into phenylalanine, aspartic acid, and methanol; the breakdown products are harmless	NAC lowers oxidative stress and cortical inflammation in the brain, which may mitigate neurotoxic effects. Elevated dosages alter the glutathione-dependent process, causing hepatic damage and affecting the liver's antioxidant capacity	Griebsch et al. [11]
Advantame	High-intensity sweetener, no sour or bitter aftertaste; stable at high and low pH levels	No teratogenic potential; no detrimental effects on progeny growth or reproduction	No possibility for teratogenicity; no detrimental effects on progeny development or reproduction in experiments on animals. Efficient at boosting the flavor of a variety of food goods without having a negative impact on humans, according to research	Ali et al. [6]
Saccharin	Poor organic acid with a specific gravity of 0.83 g/cm³, molar mass of 183.2 g/mol, and a pKa value of 1.6	Body-absorbed, attached to plasma proteins, and expelled as feces and urine	After being first connected to bladder cancer in rats, IARC and NTP research on people revealed no appreciable cancer risk. Decreases the number of anaerobic bacteria in the gut, which affects the composition of the gut microbiome	Mahmood and Al-Juboori [7]
Sucralose	Sweetener without calories	Affects insulin production; changes in gut microbiota are linked to reduced glucose tolerance	Switching from sugar-sweetened drinks to diet beverages appears to promote greater weight loss in overweight adults, according to research, and impacts mice's insulin synthesis and glucose tolerance, possibly as a result of altered gut flora	Gujral et al. [8]
Neotame	The tert-butyl group linked to modified aspartame	Aspartame-like but with more stability	Liver damage is a potential side effect of overdosing; additional symptoms include loss of appetite, minor headaches, and fluctuations in weight. Including a tert-butyl group improves its stability and contributes roughly 60% more sweetness than aspartame	Ahmad et al. [12]

Role of ASs in gut microbiota disruption and metabolic health

Dysbiosis refers to an imbalance in the microbial communities within the gut, where the normal harmony between beneficial and harmful microorganisms is disrupted. Many different types of microorganisms that are essential to immune system function, digestion, and general health reside in the gut under healthy circumstances. However, when dysbiosis occurs, harmful bacteria, yeast, or other pathogens begin to dominate, while beneficial microbes like Bifidobacterium and *Lactobacillus acidophilus* (Lactobacillus spp.) diminish. This imbalance can manifest through various gastrointestinal symptoms, including bloating, diarrhea, constipation, and abdominal pain. It may also lead to systemic issues such as inflammation and increased intestinal permeability, often termed "leaky gut." The reduction of beneficial bacteria, such as Bifidobacterium, important for breaking down dietary fiber and producing short-chain fatty acids (SCFAs) like butyrate and *L. acidophilus*, which help maintain an acidic environment in the gut, results in decreased SCFA production, compromised colon health, and a weakened gut barrier. Conversely, the proliferation of harmful bacteria like Proteobacteria, including Escherichia coli strains, can produce toxic metabolites such as lipopolysaccharides (LPS), which trigger inflammation and contribute to metabolic disorders, obesity, and chronic diseases. This overgrowth also disrupts normal gut functions, exacerbating symptoms like diarrhea, bloating, and pain.

Mechanism and Pathophysiology

**Figure 2:**
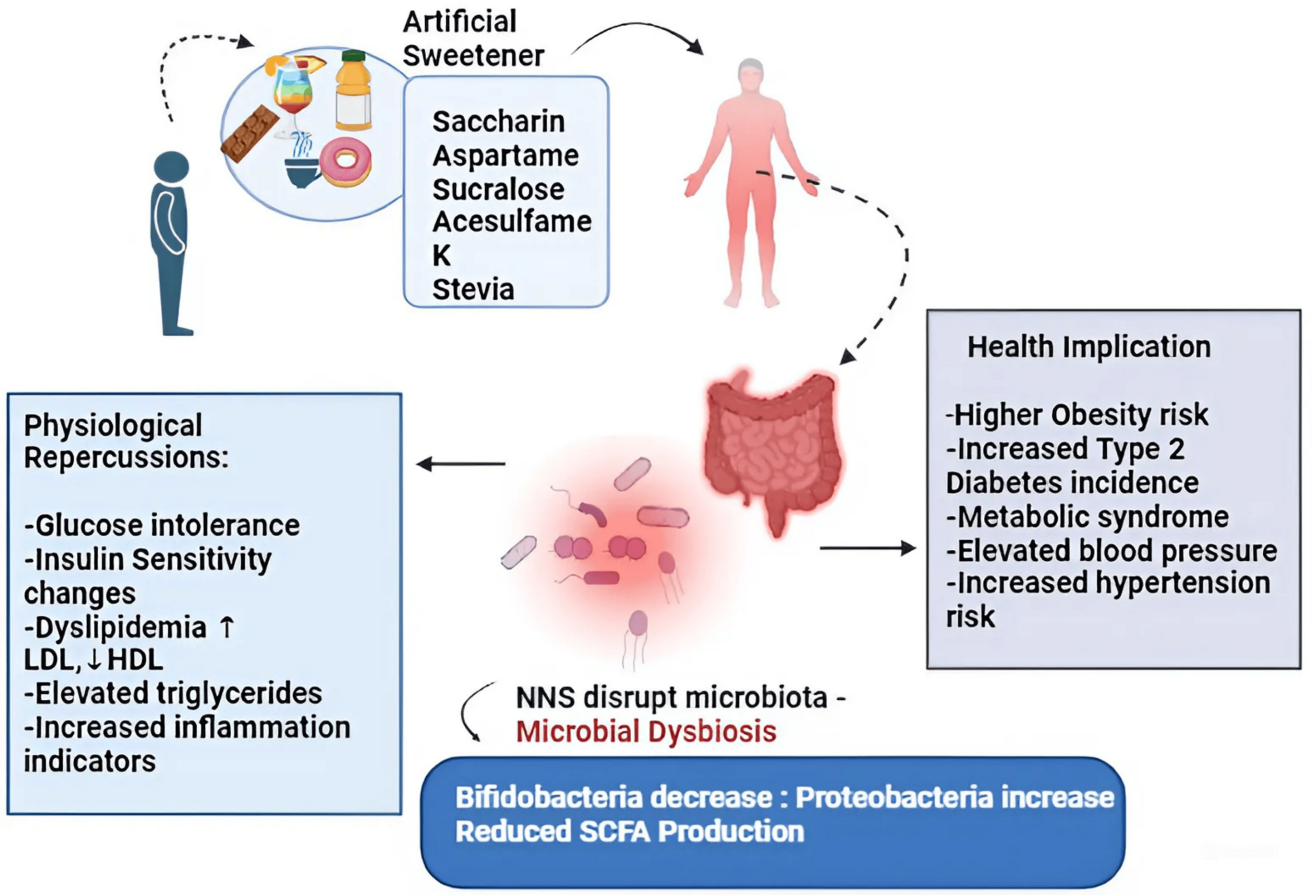
Gut microbiota dysbiosis induced by ASs, depicting how ASs like saccharin, aspartame, sucralose, acesulfame-K, and stevia impact gut microbiota, leading to microbial dysbiosis. This dysbiosis, characterized by reduced Bifidobacterium and increased Proteobacteria, lowers SCFA production and results in dyslipidemia, inflammation, glucose intolerance, and altered insulin sensitivity. These physiological changes heighten the risk of obesity, T2DM, metabolic syndrome, and hypertension LDL: low-density lipoprotein; HDL: high-density lipoprotein; NNS: nonnutritive sweeteners; ASs: artificial sweeteners; T2DM: type 2 diabetes mellitus; SCFA: short-chain fatty acid This image was illustrated by the author Meenatchi M

ASs, including saccharin, aspartame, sucralose, and acesulfame-K, can profoundly impact gut microbiota, leading to microbial dysbiosis. This dysbiosis is characterized by a reduction in beneficial bacteria such as Lactobacillus and Bifidobacterium and an increase in potentially harmful Proteobacteria. This imbalance disrupts the microbial community, resulting in decreased production of SCFAs, which are crucial for lipid and glucose metabolism. Consequently, this can contribute to glucose intolerance, dyslipidemia (elevated low-density lipoprotein, reduced right-density lipoprotein), as illustrated in Figure 2, increased triglycerides, and heightened inflammation. Such changes elevate the risk of obesity, insulin resistance, type 2 diabetes mellitus (T2DM), metabolic syndrome, and cardiovascular conditions, including hypertension. Moreover, ASs can exacerbate these issues by further disrupting the gut microbiota. Their bacteriostatic effects inhibit the growth of specific gut bacteria, altering the overall microbial environment. This disruption increases intestinal permeability, or "leaky gut," allowing toxins to enter the bloodstream and induce systemic inflammation. This inflammation impairs insulin signaling and glucose metabolism, which can contribute to the development of T2DM. High consumption of ASs has also been linked to an elevated risk of cardiovascular diseases. The disruption of glucose metabolism, increased insulin resistance, and systemic inflammation caused by gut dysbiosis contribute significantly to these risks. For instance, studies like the Women's Health Initiative have shown that excessive consumption of artificially sweetened beverages is associated with a higher risk of stroke, coronary heart disease, and mortality. Thus, the imbalance in gut microbiota caused by ASs plays a critical role in the pathogenesis of metabolic disorders and cardiovascular conditions [13].

Clinical Studies

The clinical study conducted by Wang investigated the effects of ASs, including aspartame, sucralose, and saccharin, on gut microbiota. This research involved human participants who consumed these sweeteners over controlled periods, revealing alterations in gut microbiota composition, particularly in the abundance of bacterial taxa such as Bacteroides and Firmicutes. Laboratory analyses, including 16S ribosomal RNA gene sequencing and metagenomics, demonstrated that these sweeteners disrupted the normal gut microbial balance, potentially leading to dysbiosis. Participants reported metabolic changes, such as impaired glucose tolerance, and laboratory findings highlighted reductions in beneficial SCFAs like butyrate, which are crucial for maintaining gut health. The study suggests that the consumption of ASs can negatively impact gut microbiota, with potential implications for metabolic and gastrointestinal health [14].

A study by Richardson and Frese investigated the effects of ASs on gut microbiota and metabolic health. In their research, seven participants who consumed saccharin for one week showed increased harmful bacterial taxa such as Bacteroides and Clostridia in stool samples, alongside a decrease in beneficial Lactobacilli. These microbial shifts were associated with impaired glucose tolerance and reduced insulin sensitivity, as evidenced by elevated blood glucose levels and a diminished insulin response during metabolic testing. The researchers proposed that saccharin-induced gut dysbiosis might increase intestinal permeability, allowing endotoxins to trigger systemic inflammation and disrupt insulin signaling. In a study involving 24 participants who consumed aspartame daily for 12 weeks, the researchers observed a shift in gut microbiota characterized by increased proinflammatory bacteria and decreased microbial diversity. This change was accompanied by elevated inflammatory markers, such as C-reactive protein, and mild metabolic disturbances, including higher fasting glucose levels. The study hypothesized that aspartame affects gut microbiota by promoting the growth of bacteria that produce proinflammatory compounds, leading to systemic inflammation and altered metabolic processes. These findings highlight the potential for ASs to disrupt gut microbiota and contribute to metabolic disorders, emphasizing the need for further research into their long-term health effects [15].

In a study by del Pozo et al., patients who consumed ASs such as aspartame, saccharin, and sucralose exhibited significant alterations in their gut microbial composition. The findings highlighted a decrease in beneficial bacterial strains, such as Lactobacillus and Bifidobacterium, which are essential for maintaining gut health. Additionally, an increase in bacterial strains associated with adverse metabolic outcomes, including glucose intolerance and increased body weight, was observed. These microbiome changes were correlated with clinical symptoms such as gastrointestinal discomfort, altered bowel movements, and metabolic disturbances, suggesting the potential negative impact of ASs on gut health and overall metabolic function. The study underscores the need for careful consideration of the effects of ASs on gut microbiota, especially in patients with preexisting metabolic or gastrointestinal conditions [16].

In the study by Meenakshi and Mohan, a noticeable reduction in populations of beneficial bacteria such as Lactobacillus and Bifidobacterium was reported, alongside an increase in microbial species associated with dysbiosis. These gut microbiota composition changes could explain the patient's recent symptoms, including mild glucose intolerance and gastrointestinal discomfort. Gut dysbiosis caused by ASs can impair glucose tolerance and lead to insulin resistance. Reducing SCFA production, which plays a vital role in lipid and glucose metabolism, exacerbates these metabolic dysfunctions. The increase in harmful bacteria leads to a higher production of inflammatory compounds, further disrupting insulin signaling and glucose homeostasis. The findings are consistent with current research suggesting that ASs like saccharin, sucralose, and aspartame can disrupt the gut microbial balance, potentially leading to metabolic disturbances. Given these results, it is recommended that the patient reduces their intake of ASs and undergoes further monitoring to assess the long-term effects on their gut health and overall metabolic function [17].

Preclinical Study

Animal studies have provided more insight into the mechanisms by which ASs may affect health. In rodent models, exposure to ASs has been shown to cause alterations in the gut microbiota, leading to metabolic disturbances such as impaired glucose metabolism and increased fat deposition [16,17]. The dysbiosis observed in these studies often involves a reduction in beneficial gut bacteria and an increase in harmful bacteria, similar to what has been observed in humans. These changes in the gut microbiota may contribute to the development of metabolic disorders and cardiovascular diseases, reinforcing the findings from clinical studies.

Health conditions linked to dysbiosis

**Figure 3:**
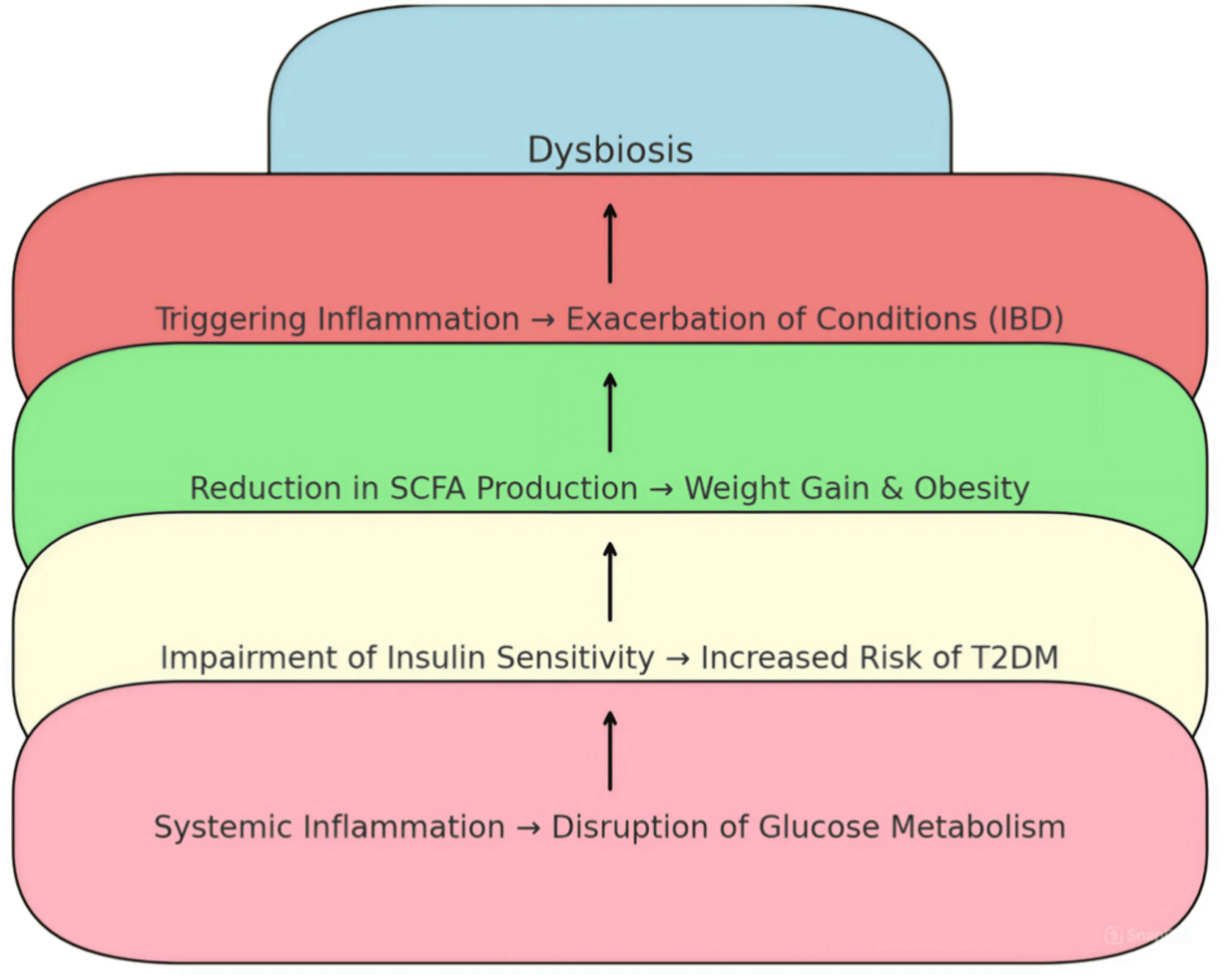
Impact of dysbiosis on health, showing ASs can disrupt gut microbiota, leading to an imbalance that increases harmful bacteria and impairs gut barrier function. This can result in systemic inflammation, potentially worsening conditions like IBD, promoting weight gain and obesity, and increasing the risk of T2DM by impairing insulin sensitivity and disrupting glucose metabolism IBD: inflammatory bowel disease; SCFA: short-chain fatty acid; T2DM: type 2 diabetes mellitus; ASs: artificial sweeteners This image was illustrated by the author Meenatchi M

Figure 3 demonstrates the cascading impact of dysbiosis, an imbalance in gut microbiota, on overall health. Dysbiosis is shown as the starting point, leading to various negative health outcomes. Initially, it triggers inflammation, which can worsen conditions like IBD. Next, it reduces the production of SCFAs, contributing to weight gain and obesity. Dysbiosis also impairs insulin sensitivity, increasing the risk of T2DM. Ultimately, the systemic inflammation caused by dysbiosis disrupts glucose metabolism, leading to broader metabolic issues. The figure highlights the complex relationship between gut and systemic health, demonstrating the wide-reaching effects of dysbiosis.

Dysbiosis and IBD

Mechanism and Pathophysiology

**Figure 4:**
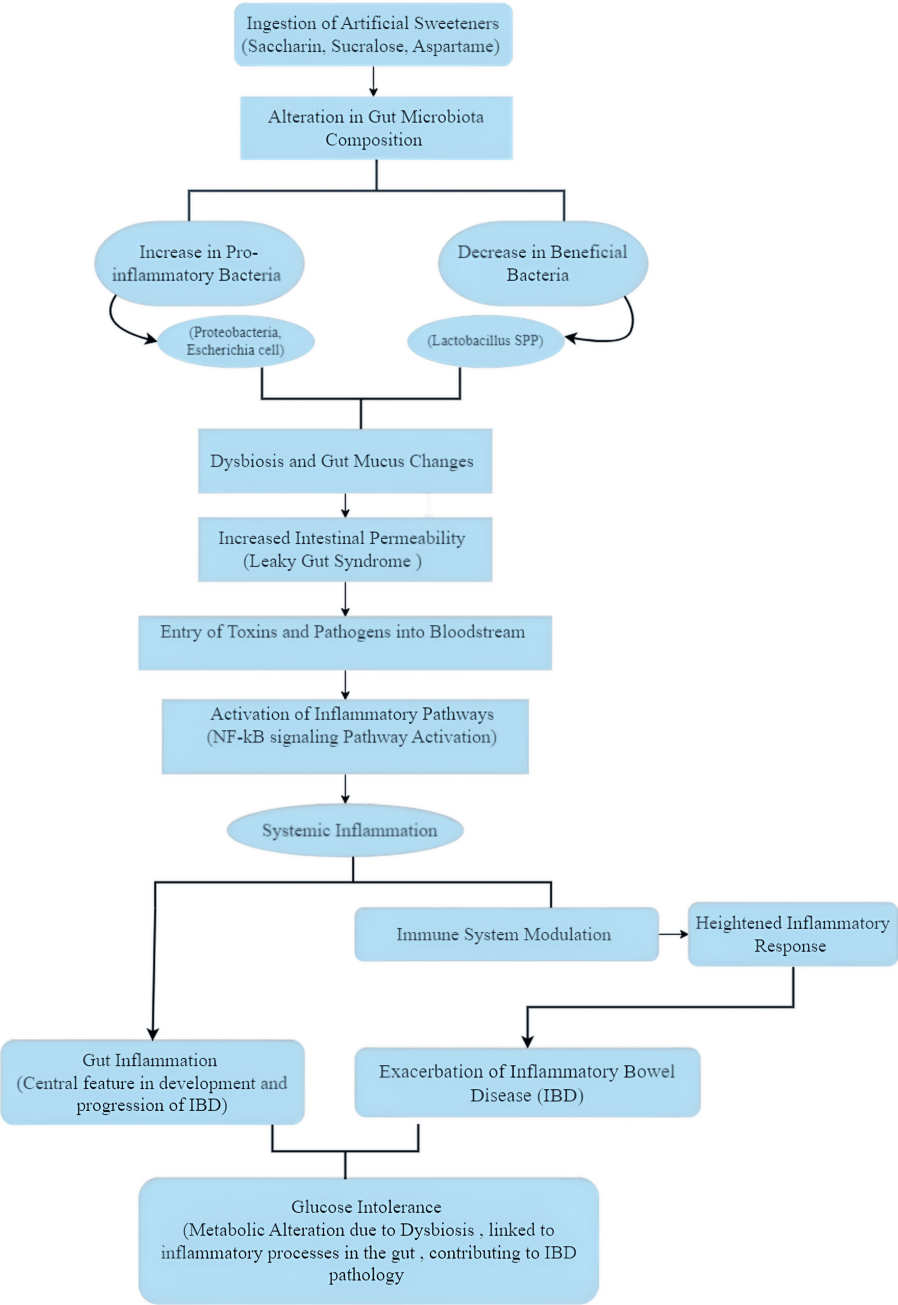
Impact of ASs on gut health and inflammation, depicting how ASs like saccharin, sucralose, and aspartame can alter gut microbiota, increasing proinflammatory bacteria and reducing beneficial ones. This imbalance (dysbiosis) leads to "leaky gut syndrome," allowing toxins into the bloodstream and triggering inflammation through the NF-κB pathway. The resulting systemic inflammation can worsen IBD and cause glucose intolerance, further contributing to IBD pathology ASs: artificial sweeteners; NF-kB: nuclear factor kappa B; IBD: inflammatory bowel disease This image was illustrated by the author Meenatchi M

ASs, including saccharin and sucralose, have been shown to significantly affect gut health by interacting with the gut microbiota. These sweeteners alter the gut flora composition, promoting dysbiosis characterized by an increase in proinflammatory bacteria such as Proteobacteria and *E. coli* while simultaneously reducing beneficial bacteria like Lactobacillus spp. [18]. Dysbiosis, coupled with changes in the gut's mucus structure, can increase intestinal permeability, leading to "leaky gut syndrome." This condition allows toxins and pathogens to pass into the bloodstream, triggering inflammatory pathways and contributing to systemic inflammation and the exacerbation of IBD [19]. Furthermore, ASs have been found to modulate immune responses, worsening gut inflammation. Specifically, saccharin increases the presence of Bacteroides species while reducing Lactobacillus, fostering a proinflammatory state. This dysbiotic environment activates the nuclear factor kappa B (NF-κB) signaling pathway, a key promoter of inflammation, as shown in Figure 4 [20]. The combination of these gut microbiota changes and increased intestinal permeability can lead to gut inflammation, a hallmark of IBD pathology. Prolonged consumption of ASs can also contribute to metabolic dysregulation, such as glucose intolerance, exacerbating inflammatory processes and linking sweeteners to both metabolic and inflammatory diseases like IBD [19,20].

Clinical Study

The study by Rodriguez-Palacios et al. investigated the effects of the AS Splenda on patients with Crohn's disease (CD). In 19 CD patients and 20 healthy controls, the study revealed that Splenda, which contains sucralose and maltodextrin, significantly affected CD patients' inflammatory markers and gut microbiota. Specifically, using Splenda led to increased myeloperoxidase (MPO) reactivity, with elevated MPO levels observed in the stool samples of CD patients consuming Splenda compared to those who did not. The study also noted a notable rise in Proteobacteria in the gut microbiota of these patients, which is associated with inflammation. Additionally, there was increased bacterial infiltration into the ileum among CD patients using Splenda. These findings underscore the potential exacerbation of CD symptoms due to ASs, highlighting the need for caution in their consumption by individuals with IBD [21].

The study by Basson et al. investigated the impact of ASs on individuals with IBD by following 100 IBD patients for six months. The study found that those consuming ASs experienced a significant increase in gastrointestinal symptoms, including abdominal pain, diarrhea, and bloating, compared to those avoiding ASs. Laboratory analyses also revealed a decrease in beneficial gut bacteria such as Bifidobacteriumand an increase in harmful bacteria like Firmicutes in the AS group, suggesting that ASs may exacerbate IBD symptoms by disrupting gut microbiota and promoting intestinal inflammation [22].

The study by Adolph and Zhang investigates how dietary factors, including ASs like saccharin and sucralose (Splenda), affect the incidence of IBD. The study's findings indicate that these sweeteners may contribute to gut dysbiosis, which is defined by an increase in Bacteroidesspecies and a decrease in Lactobacillus species, ultimately resulting in elevated inflammation. Furthermore, it emphasizes how Splenda exacerbates intestinal inflammation, leading to an overabundance of Proteobacteria and *E. coli*, both of which are connected to aggravating IBD. A prospective trial headed by Levine et al. that involved 78 pediatric CD patients is also mentioned in the report. In a 12-week trial, the CD exclusion diet in combination with partial enteral nutrition was found to be more effective and well-tolerated than exclusive enteral nutrition. This resulted in better disease control and higher rates of remission [23].

The study by Dai et al., conducted in Sweden by Khalili et al., explored the relationship between ASs, such as saccharin and sucralose, and the risk of developing CD and ulcerative colitis. Although the exact number of participants was not provided, such studies typically involve thousands to ensure statistical significance. The findings revealed no significant association between the consumption of sweetened beverages and the later risk of developing these conditions, contradicting earlier research suggesting that ASs might contribute to IBD pathogenesis by affecting gut bacteria and digestive protease inactivation. The study highlighted the need for further research into the role of intestinal microbiota and digestive proteases, with future lab tests likely focusing on gut microbiota analysis, protease activity assays, and inflammation markers [24].

Preclinical Study

Animal studies have provided significant insights into the effects of ASs on gut health and behavior. For example, research on Splenda has shown that it increases gut inflammation in senescence-accelerated mouse prone stains by promoting the overgrowth of harmful bacteria such as *E. coli* and Proteobacteria. This bacterial imbalance, known as dysbiosis, leads to a rise in proinflammatory bacteria, which disrupts the mucus architecture of the gut and contributes to inflammation. Similarly, saccharin consumption in mice has been found to alter gut microbiota by increasing the levels of Bacteroides and reducing Lactobacillus species, further promoting dysbiosis and gut inflammation. Additionally, aspartame has been shown to impact social behavior, object-directed behavior, and cognitive function in rats, with these behavioral changes potentially linked to alterations in gut microbiota composition [21].

Dysbiosis and obesity

Mechanism and Pathophysiology

**Figure 5:**
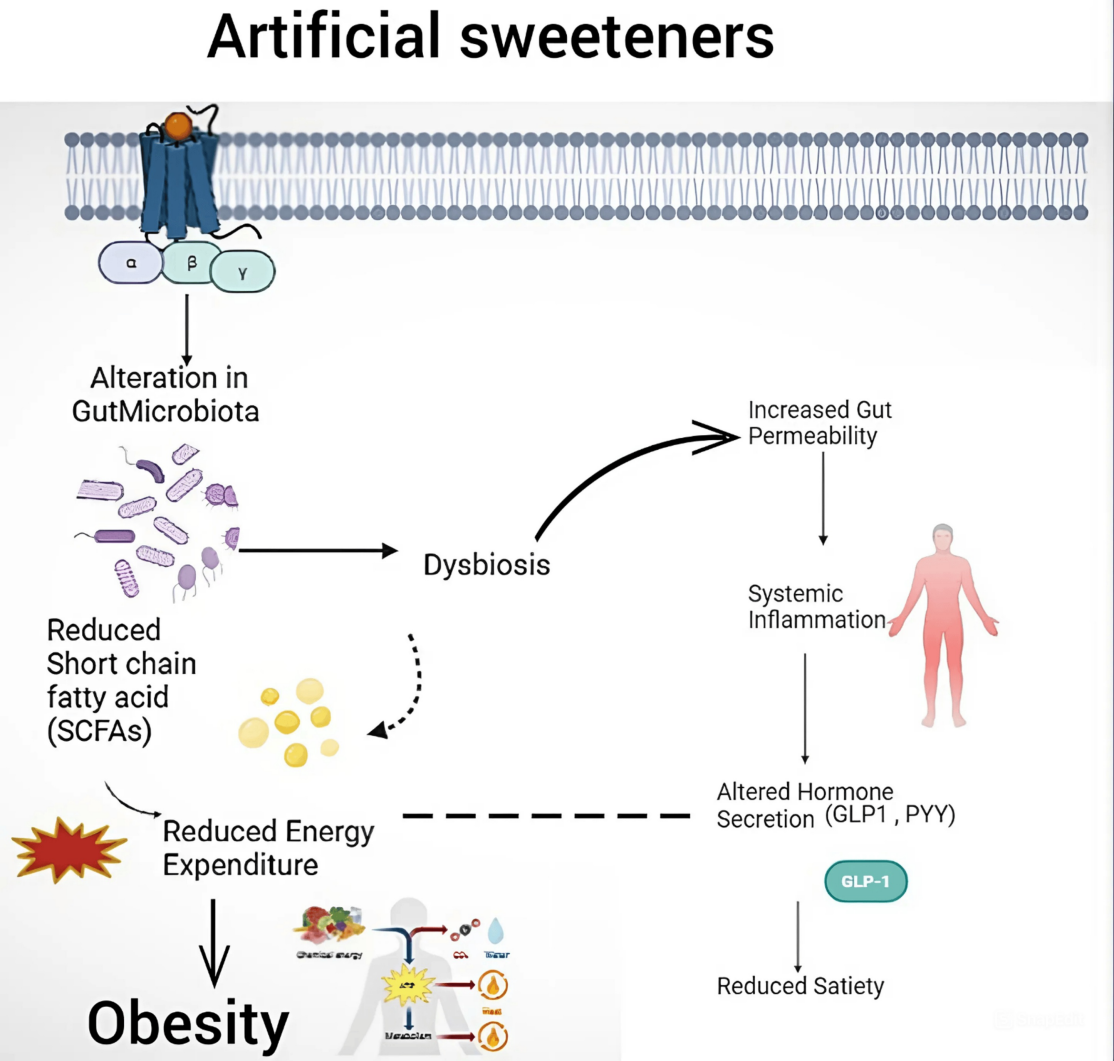
Effects of ASs on obesity and gut health. ASs can alter gut microbiota, leading to dysbiosis, reduced SCFA production, and increased gut permeability. This imbalance raises systemic inflammation, disrupts hunger and satiety hormones (GLP-1 and PYY), and contributes to overeating and obesity ASs: artificial sweeteners; SCFA: short-chain fatty acid; GLP-1: glucagon-like peptide-1; PYY: peptide tyrosine tyrosine This image was illustrated by the author Meenatchi M

ASs, such as saccharin and sucralose, can significantly impact gut health by altering the composition of gut microbiota, leading to a condition known as dysbiosis. This dysbiosis reduces beneficial SCFAs, which are crucial for maintaining energy metabolism and supporting gut barrier function. Consequently, the body experiences reduced energy expenditure, contributing to weight gain and obesity. Moreover, the altered gut microbiota increases intestinal permeability, often called "leaky gut." This condition allows harmful substances, including toxins and bacteria, to enter the bloodstream, triggering systemic inflammation. The inflammatory response further exacerbates metabolic dysfunction by affecting the secretion of key hormones such as glucagon-like peptide-1 (GLP-1) and peptide tyrosine tyrosine illustrated in** **Figure 5, which play vital roles in regulating hunger and satiety. Disrupted hormone secretion leads to decreased feelings of fullness, resulting in overeating and the potential development of obesity. This cascade of events underscores the complex interplay between ASs, gut microbiota, and metabolic health, indicating that AS consumption can have far-reaching consequences beyond simple calorie reduction. The interaction between these factors suggests that the impact of ASs on metabolic pathways is multifaceted, contributing not only to obesity but also to broader metabolic disorders. Understanding these mechanisms highlights the importance of considering individual gut microbiome responses when assessing the metabolic risks associated with ASs [25].

Clinical Study

A comprehensive study conducted by Harrold et al. involving 47,910 adults from 11 diverse cohorts examined the relationship between high-intensity ASs and obesity. Aspartame, saccharin, sucralose, and stevia were among the sweeteners examined; 24-hour dietary recalls and food frequency questionnaires were used to gather consumption data. The study discovered, throughout a four-to-seven-year follow-up period, a substantial correlation between high aspartame intake and an elevated risk of obesity. This correlation held true for all age groups and baseline body mass index (BMI) levels. Sucralose and saccharin also showed a positive, albeit less pronounced, association with obesity, while stevia did not appear to be linked to obesity risk. Although other factors, including overall food quality and physical activity levels, may have an impact, the results indicate that heavy use of some ASs, especially aspartame, may lead to weight gain and obesity [26].

In the study examined by Pearlman et al., the effects of ASs on obesity consistently show a link between their consumption and increased BMI and adiposity. For example, they referenced Azad et al., who found that in a cohort of 3,033 mothers, daily intake of artificially sweetened beverages during pregnancy was associated with a twofold increase in the risk of their infants being overweight by one year of age. Additionally, Pepino et al.'s studies observed that AS consumption could lead to weight gain by promoting higher peak insulin levels and altering metabolic responses. Research also suggests that ASs may contribute to weight gain through mechanisms like increased calorie intake and disruptions in the gut microbiome. Despite being marketed as a healthier alternative to sugar, the evidence indicates that ASs may promote obesity, highlighting the complexity of their effects on weight management [27].

The prospective study conducted by Debras et al. involving 103,388 individuals tracked for an average of 7.8 years to assess the relationship between the use of ASs and the risk of obesity. The results showed a correlation between an elevated risk of obesity and higher usage of ASs, including aspartame, acesulfame-K, and sucralose. In particular, compared to individuals with lower intake, those in the highest quartile of AS intake showed a hazard ratio (HR) of 1.09 (95% confidence interval, 1.03-1.16) for obesity. Based on their effects on hunger and metabolism regulation, ASs may raise the risk of obesity even when they are used as sugar alternatives for weight control [28].

Preclinical Study

A study was conducted to investigate the effects of ASs on obesity in male Wistar rats, focusing on body weight, glucose levels, insulin, and leptin. The rats were divided into 18 groups, with nine groups fed a control diet and the other nine a high-fat (HF) diet, each group receiving different ASs in their drinking water, including sucrose, steviol glycosides, and sucralose. The results showed that sucrose, sucralose, and steviol glycosides led to significantly higher body weights, especially when combined with the HF diet, which further elevated serum glucose, insulin, and leptin levels. Notably, sucralose-fed rats had the highest glucose (151.5 mg/dL), insulin (6.44 ng/mL), and leptin (25.54 ng/mL) levels, indicating a pronounced impact on obesity-related parameters. This study suggests that ASs, particularly sucralose and sucrose, may exacerbate obesity, especially in the context of HF diets [29].

A study on the AS neotame investigated its impact on obesity-related factors in CD-1 male mice, revealing significant alterations in the gut microbiome and fecal metabolites. Over four weeks of neotame treatment, the mice exhibited reduced gut microbiome diversity and notable dysbiosis, with an increased proportion of Bacteroidetes and a decrease in Firmicutes. This change was accompanied by a decline in butyrate fermentation, an essential mechanism for gut health, and a reduction in beneficial gut bacteria. Significant alterations in fecal metabolites, such as a reduction in essential fatty acids and a rise in cholesterol, were also revealed by metabolomic analysis. These findings suggest that neotame consumption can disrupt gut microbiota and metabolic processes, potentially contributing to obesity [30].

Dysbiosis and T2DM

Mechanism and Pathophysiology

**Figure 6:**
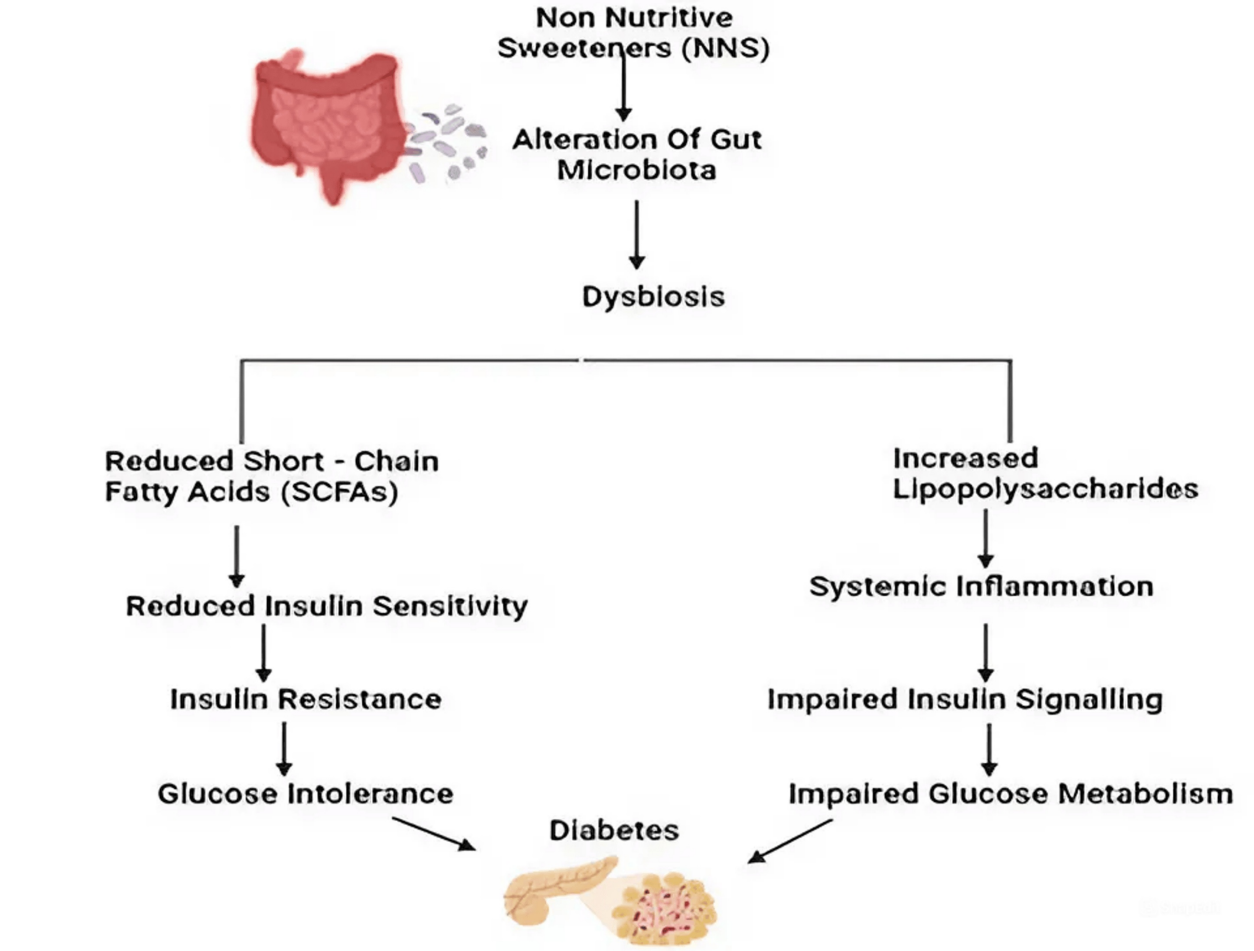
Impact of nonnutritive sweeteners on gut microbiota and diabetes development, depicting how ASs disrupt gut microbiota, leading to dysbiosis. This imbalance reduces short-chain fatty acids, lowering insulin sensitivity and causing insulin resistance, which can lead to glucose intolerance and diabetes. Dysbiosis also increases LPS, triggering systemic inflammation, impaired insulin signaling, and further disrupting glucose metabolism, thereby increasing the risk of diabetes ASs: artificial sweeteners; LPS: lipopolysaccharides This image was illustrated by the author Meenatchi M

ASs influence glucose metabolism and insulin resistance through several pathways, as illustrated in Figure 6. Consumption of AS alters the gut microbiota, leading to dysbiosis. This imbalance in the gut flora reduces SCFAs, which is crucial for maintaining insulin sensitivity. The decrease in SCFAs leads to reduced insulin sensitivity, causing insulin resistance and glucose intolerance, ultimately contributing to the development of T2DM, as illustrated in Figure 6. Additionally, dysbiosis triggers an increase in LPS, which promotes systemic inflammation, impairs insulin signaling, and disrupts glucose metabolism. Together, these processes enhance the risk of diabetes by adversely affecting insulin signaling and glucose homeostasis. ASs can also stimulate the release of gut hormones like GLP-1 and gastric inhibitory polypeptide, further impacting glucose homeostasis. Prolonged AS consumption may overstimulate pancreatic cells, leading to hyperinsulinemia and potential long-term pancreatic dysfunction. The interplay between altered gut microbiota, systemic inflammation, and pancreatic function highlights the complex mechanisms through which ASs contribute to metabolic disorders like diabetes [31].

Clinical Studies

The study conducted by Debras et al., involving 105,588 participants and a median follow-up of 9.1 years, was used to examine the effect of ASs on the risk of T2D; 38.1% of participants in the study reported using ASs, with an average intake of 100 mL/day. Nine hundred seventy-two T2D event instances occurred during the study period. An increased risk of T2DM was found to be significantly correlated with higher usage of ASs. The study found that the HRs for aspartame, acesulfame-K, sucralose, and total AS intake were 1.69, 1.63, 1.70, and 1.34, respectively. The findings raise questions regarding the safety of ASs as sugar substitutes because they imply that aspartame, acesulfame-K, and sucralose may increase the incidence of T2DM [28].

The potential link between the use of ASs and the onset of T2DM was examined by a comprehensive review and meta-analysis. The combined analysis of 18 papers, totaling more than 400,000 individuals, showed a strong positive correlation with a relative risk of 1.12 per daily serving between the consumption of ASs and the risk of T2DM. To be more precise, sucralose had no significant correlation with risk, whereas aspartame and saccharin were linked to 1.17- and 1.15-fold higher risks, respectively. Some research also revealed further laboratory results that suggested altered gut microbiota composition, heightened insulin resistance, and impaired glucose metabolism as possible pathways connecting ASs to metabolic dysfunction. Using these metabolic pathways, these results imply that ASs may contribute to the development of T2DM [31].

According to Alsunni's study, which involved 17 obese adults, consuming sucrose resulted in a 20% increase in insulin levels and a 23% decrease in insulin sensitivity but no discernible change in fasting glucose levels. In a bigger trial involving 66 healthy participants, sucrose was found to modestly reduce insulin sensitivity by 10% while having no effect on fasting glucose or HbA1c levels. However, during the course of a 12-week trial involving 48 subjects, including T2D patients, aspartame was found to have no discernible impact on fasting glucose, insulin sensitivity, or HbA1c levels. Another study, including 20 healthy adults, discovered that aspartame did not affect insulin sensitivity, glucose tolerance, or GLP-1 levels. Finally, a study of 30 obese individuals who regularly consumed diet sodas found no significant impact of mixed ASs on glucose tolerance, insulin response, or HbA1c levels. Overall, the evidence suggests that while sucralose may slightly affect insulin sensitivity, the overall impact of ASs on diabetes risk remains inconclusive [32].

De la Hunty et al. (2010) investigated the impact of AS on glucose metabolism and the risk of developing diabetes by a comprehensive review and meta-analysis. Observational studies that included over 160,000 female participants in the Nurses' Health Studies I and II and over 40,000 male participants in the Health Professionals Follow-Up Study first discovered strong links between artificially sweetened beverages and T2DM. However, these relationships frequently became less significant when controlling for energy intake and BMI. Similarly, a correlation between artificially sweetened drinks and diabetes was found in the European Prospective Investigation into Cancer and Nutrition trial, involving 340,234 men and women from all around Europe. However, this correlation also decreased with changes in BMI. More specific information was obtained from clinical trials. For example, sucralose was found to raise glucose concentrations, reduce insulin sensitivity, and elevate insulin and C-peptide levels in people with an average BMI of 42 kg/m^2^. Another trial by Suez et al. involving seven subjects suggested that saccharin consumption could negatively impact glucose tolerance by altering the intestinal microbiome. Overall, while some studies indicate a potential risk of diabetes associated with ASs, the evidence is complex and often influenced by other factors like BMI [33].

Preclinical Study

Animal studies investigating the risk of diabetes associated with ASs employed various methods and yielded significant findings. One study used isolated perfused pancreas from rats to examine the impact of sweeteners like saccharin, sucralose, and acesulfame-K on insulin secretion. While physiological doses (50 µM) had no significant effect, higher doses (50 mM) increased insulin secretion, particularly with acesulfame-K showing the highest potency. Insulin secretion was mediated by activating sweet taste receptors, with acesulfame-K > saccharin = sucralose in potency. Additionally, intestinal glucose absorption was studied in wild-type mice and T1R3 knockout mice. Sweeteners such as sucralose (2 mM) increased sodium-glucose cotransporter 1 mRNA and protein expression, enhancing glucose absorption in wild-type mice but not in knockout mice. Further, in rats, both acesulfame-K and sucralose increased glucose absorption and intracellular calcium concentrations, with sucralose (4%) and saccharin stimulating glucagon-like peptide-2 secretion. Daily consumption of saccharin, sucralose, and aspartame led to glucose intolerance in mice within a week, with saccharin particularly exacerbating HF diet-induced glucose intolerance. This effect was linked to the gut microbiota, as antibiotic treatment mitigated glucose intolerance. These findings suggest that at higher doses, ASs can disrupt glucose metabolism, potentially increasing the risk of diabetes, with significant changes observed in insulin secretion, glucose absorption, and glucose tolerance in animal models [34].

Discussion

The discussion highlights the complexities surrounding ASs, which are widely used as low-calorie sugar substitutes but are linked to various health risks. Despite their approval for managing conditions like diabetes and obesity, emerging research suggests potential associations with metabolic disorders, cardiovascular disease, and cancer. High consumption of these sweeteners has been linked to increased risks of stroke, heart disease, mortality, weight gain, and adverse pregnancy outcomes. Additionally, concerns about their impact on gut health are significant, with studies indicating links to IBDs and disruptions in gut flora. Regarding regulatory oversight, the ADI levels for these sweeteners are set at 50 mg/kg body weight per day for aspartame in the United States and 40 mg/kg in the European Union. In comparison, saccharin has an ADI of 5 mg/kg body weight. Based on current evidence, these thresholds aim to ensure safety. However, the ongoing health concerns and emerging research highlight the need for informed consumer choices and stringent regulation. Regulatory bodies like the EFSA continue to review these sweeteners, underscoring the necessity for updated guidelines and public health recommendations [1-12]. ASs have been increasingly scrutinized for their role in gut microbiota disruption and the subsequent effects on metabolic health. Dysbiosis, or the imbalance of gut microbiota, is a central concern, as it disrupts the delicate harmony between beneficial and harmful microorganisms within the gut. This imbalance can lead to various health issues, including gastrointestinal symptoms, systemic inflammation, and metabolic disorders like obesity and T2DM.

Under normal conditions, the gut is home to diverse microbial communities that play critical roles in digestion, immune function, and overall health. Beneficial bacteria, such as Bifidobacteriumand Lactobacillus spp., are essential for breaking down dietary fibers and maintaining gut barrier integrity. However, dysbiosis results in the proliferation of harmful bacteria, such as Proteobacteria and *E. coli*, while reducing the population of beneficial microbes. This leads to decreased SCFA production, compromised gut health, and increased intestinal permeability, often called "leaky gut." The entry of toxins into the bloodstream triggers systemic inflammation, contributing to the development of chronic diseases like obesity, insulin resistance, and cardiovascular conditions. ASs, including saccharin, aspartame, sucralose, acesulfame-K, and stevia, have been shown to impact gut microbiota significantly. These sweeteners can induce dysbiosis by reducing beneficial bacteria and promoting the growth of harmful ones. This microbial imbalance can result in impaired glucose metabolism, dyslipidemia, and heightened inflammation, all of which are risk factors for obesity and metabolic syndrome. The bacteriostatic effects of nonnutritive sweeteners further exacerbate these issues by inhibiting the growth of specific gut bacteria, leading to altered microbial communities and increased oxidative stress [13].

Several clinical studies have investigated the impact of ASs on gut microbiota and metabolic health. For example, Wang demonstrated that ASs disrupt the gut microbiota, leading to impaired glucose tolerance and reduced SCFA production. Similarly, Richardson and Frese found that saccharin consumption increased harmful bacteria and decreased beneficial Lactobacilli, impairing glucose tolerance and systemic inflammation [14,15].

Preclinical studies in rodent models have provided further insights into the mechanisms by which ASs affect gut health. These studies have shown that exposure to ASs can lead to dysbiosis, resulting in metabolic disturbances like impaired glucose metabolism and increased fat deposition. These findings reinforce the conclusions drawn from human studies, suggesting that ASs can contribute to the development of metabolic disorders [16,17].

Dysbiosis induced by ASs is also linked to obesity. Reduced SCFA production and increased gut permeability contribute to systemic inflammation and disrupted hormone secretion, leading to overeating and weight gain. Clinical studies, such as those by Harrold et al. and Debras et al., have found significant associations between AS consumption and an increased risk of obesity. These studies suggest that the impact of ASs on metabolic health is multifaceted, affecting gut microbiota, energy metabolism, and hormonal regulation [26,29].

ASs also play a role in the development of T2DM by disrupting glucose metabolism and insulin sensitivity. Dysbiosis reduces SCFA production, leading to insulin resistance and glucose intolerance. The increased production of LPS due to dysbiosis triggers systemic inflammation, further impairing insulin signaling and glucose metabolism. The complex interplay between gut microbiota, inflammation, and pancreatic function highlights the potential of ASs to contribute to the development of metabolic disorders like T2DM [31]. ASs pose several public health risks, particularly for pregnant women and individuals with metabolic disorders, as they may affect fetal development and exacerbate existing conditions. Their impact on gut health, including potential microbiome disruption and digestive issues, raises concerns. Long-term effects, such as potential links to obesity and metabolic disorders, are still under investigation. Stricter regulation, increased consumer education, and promoting natural sweeteners like stevia could mitigate these risks. Ongoing research and postmarket surveillance are essential to understanding and managing the health implications of ASs [35].

## Conclusions

In conclusion, this review underscores the health risks of ASs, including their impacts on inflammation, gut microbiota, and metabolism. While they offer a low-calorie sugar alternative, substances like aspartame, saccharin, and sucralose may cause dysbiosis, glucose intolerance, and systemic inflammation, potentially compromising metabolic health. Current ADI levels set by regulatory agencies like the FDA and EFSA aim to ensure safety but may need to be revised, particularly for high-risk groups such as pregnant women and individuals with metabolic disorders. Increased consumer awareness and consideration of natural sweeteners like stevia, which have fewer adverse effects, are recommended. As ASs become more popular, caution is advised, especially for those at risk of metabolic disorders. Enhanced public awareness and regulatory oversight are crucial. Future research is needed to fully understand the long-term effects and to develop strategies to mitigate risks. Public health policies should focus on stricter labeling, daily limits, and comprehensive awareness campaigns to manage potential health risks.
